# Comparative efficacy of supra-scapular nerve block, posterior shoulder capsule hydro-dilatation, and shoulder interval hydro-dilatation in managing shoulder adhesive capsulitis

**DOI:** 10.1007/s10067-025-07534-5

**Published:** 2025-06-18

**Authors:** Ahmed Elsaman, Shrouk Abdelmageed, Osama Sayed Daifallah

**Affiliations:** Department of Rheumatology, Faculty of Medicine, Sohag University, Sohag, Egypt

**Keywords:** Adhesive capsulitis, Musculoskeletal ultrasound, Shoulder injection

## Abstract

**Aim of work:**

This study aimed to compare the effects of supra-scapular nerve block, posterior intra-articular hydro-dilatation, and hydro-dilatation of the shoulder interval, in terms of improving pain, function, and range of motion in patients with adhesive capsulitis.

**Patients and methods:**

A total of 50 patients diagnosed with adhesive capsulitis were randomly divided into three groups. The first group received a suprascapular nerve block, the second group underwent posterior intra-articular hydro-dilatation, and the third group underwent shoulder interval hydro-dilatation. Patient assessment was conducted using visual analogue scale for pain, shoulder pain and disability index, and measurements of range of motion.

**Results:**

Group 1 experienced rapid and sustained pain reduction (*p*-value < 0.001 at both baseline vs. first follow-up and baseline vs. second follow-up), with non-significant improvement in internal rotation after 12 weeks (*p* value = 0.330). Group 2 showed delayed improvement in internal rotation (*p*-value = 0.068), but more sustained pain reduction (*p*-value < 0.001) and improved range of motion in all directions at the 12-week mark. Group 3 exhibited rapid pain reduction (*p*-value < 0.001) and improved range of motion, but non-significant improvements in internal (*p*-value = 0.131) and external rotation (*p*-value = 0.052) after 12 weeks.

**Conclusion:**

Although no significant differences were observed among the three groups, we recommend posterior intra-articular hydro-dilatation as it yielded the most promising and sustainable outcomes as regard pain reduction and range of motion improvement. Suprascapular nerve block is recommended for patients with prominent pain symptoms. Rotator interval hydro-dilatation is the least recommended intervention, being the most challenging and painful technique, and as it demonstrated a less sustained effect on range of motion.

**Table:** 

**Key Points** • *This study aimed to find the best treatment modality for adhesive capsulitis through comparing the effects of suprascapular nerve block, posterior intra-articular hydro-dilatation, and hydro-dilatation of the shoulder interval*. • *Although no significant differences were found among three modalities, posterior intra-articular hydro-dilatation showed the best long-term outcomes for pain and range of motion. Suprascapular nerve block is recommended for patients with severe pain, while hydro-dilatation of the shoulder interval had less lasting benefits*.

## Introduction

Adhesive capsulitis or frozen shoulder is a chronic inflammatory condition associated with fibrosis of the glen humeral joint that leads to gradual and progressive loss of range of movement of the shoulder, both active and passive [1].

However, it is a self-limited condition that usually has a slow recovery phase, which is satisfying in most cases even though this may take up to 2 to 3 years. The incidence of adhesive capsulitis in the general population is approximately 2 to 5% of the general population, and the incidence increases in patients with diabetes and thyroid disease [2].

Idiopathic adhesive capsulitis often involves the non-dominant extremity, although bilateral involvement has been reported in up to 40 to 50% of cases [3, 4]. The exact etiology of frozen shoulder has not been identified yet. However, there are several associated risk factors: diabetes mellitus (20%), hypothyroidism, stroke, and shoulder injury [5–7].

Contracture and decreased volume of the glen humeral capsule is the hallmark of adhesive capsulitis [5]. However, much of the disease process involves structures outside the shoulder joint including the coracohumeral ligament, rotator interval, subscapularis musculotendinous unit, and the subacromial bursae [8].

There are many options when it comes to the treatment of adhesive capsulitis, both surgical and non-surgical. Common nonsurgical treatments include medication as (NSAIDs), physical therapy, exercise, manipulation under anesthesia, steroid injection, hydro-dilatation, or nerve blockers. Surgical methods include open or arthroscopic capsular release, which improve the shoulder range of motion and alleviate pain but leave other complications. Persistent pain and limited motion despite 3 to 6 months of conservative treatment should be taken into account in the choice of surgical treatment.

All of these modalities are safe and effective in improving pain and range of motion in patients with adhesive capsulitis. In a systematic review of Jie Zhang et al. compared the efficacy of different non-surgical treatment strategies, they found that capsular distension and extracorporeal shockwave therapy showed the highest ranking for pain relief and functional improvement, respectively. Laser therapy also showed benefits for pain relief [9].

Another systematic review by Dimitris et al. also aimed at answering the question “Are any treatment modalities for frozen shoulder associated with better outcomes than other treatments?” Their study revealed that the early use of IA corticosteroid in patients with frozen shoulder of less than 1-year duration is associated with better outcomes [10].

However, while there are many approaches to treatment of adhesive capsulitis, systematic reviews of previous literature indicate mixed reports over which treatment yields the most beneficial results [11]. Or what are the factors that physicians should consider while tailoring the best treatment modality for each patient, such as age, disease duration, the most prominent symptom—whether pain or limitation of motion—and the etiology. Therapeutic management of adhesive capsulitis still varies among specialists based on personal experience instead of published evidence, that is the reason why this study aimed to compare between the effects of three treatment modalities: supra-scapular nerve block, hydro-dilatation of the shoulder capsule, and hydro-dilatation of shoulder interval as regard improvement of pain and range of motion in patients diagnosed with adhesive capsulitis.

## Patients and methods

### Patients

The study enrolled a cohort of 50 patients who had been clinically diagnosed with adhesive capsulitis, a condition characterized by significant limitations in passive shoulder external rotation, disruptive pain during daily activities, and restricted active and passive movement to less than a hundred degrees. The sample size was calculated according to the equation [12]:

$$N=z^2\;p\;\left(1-p\right)/d^2$$

where

*N*: the desired sample size.
Z: the statistic corresponding to level of confidence (1.96).
*P*: expected prevalence or proportion (0.03) [13]
*d*: precision (*d* is considered 0.05 to produce good precision and smaller error of estimate).
*N*: 1.96^2^ × 0.03 (1 − 0.03)/0.05^2^ = 45.

The sample size was increased to be 50 cases to avoid non-response rate and dropouts.

**Fig. 1:**
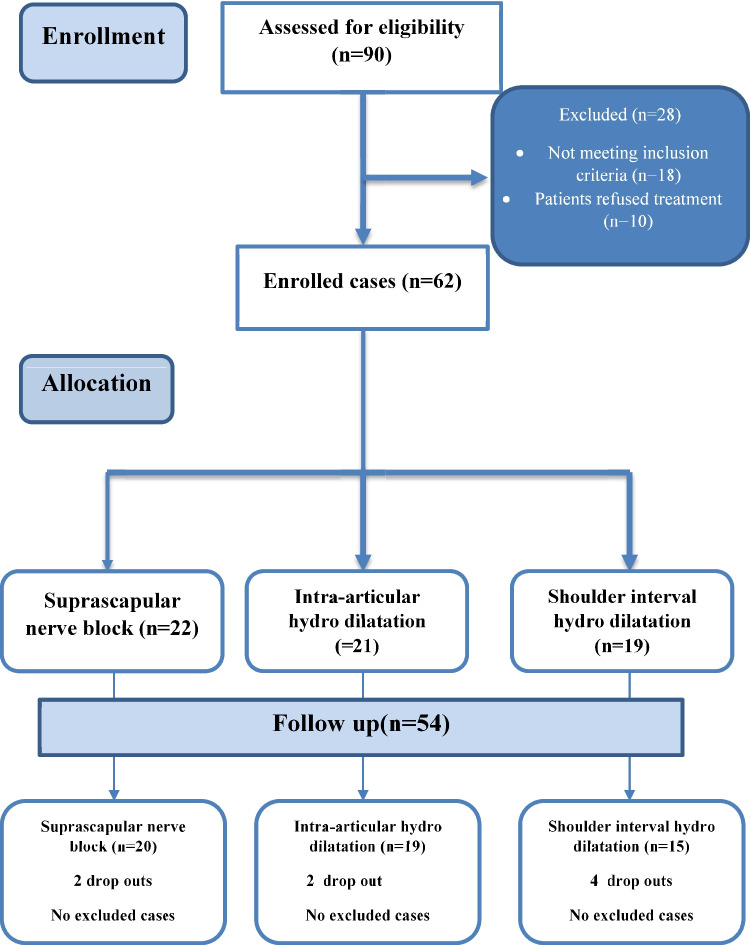
CONSORT flowchart of the enrolled patients

Four of these patients had bilateral shoulder injection, and all were included in the study, which increased the number to 54. The inclusion criteria required patients to be over 16 years of age, while exclusion criteria included fractures, psychiatric disorders, and known allergies to lidocaine. A flow diagram showing the process of patient selection is illustrated in Fig. 1**.**

To ensure unbiased allocation, a randomization process was implemented using three cards for every three patients. Each card represented one of the three intervention types under investigation. The first patient blindly selected a card, followed by the second patient, and finally, the third patient received the remaining card. Patients were unaware of the type of intervention they have chosen, as the cards contained only numbers, number 1 referred to suprascapular nerve block, 2 referred to posterior intra articular hydro-dilatation, and 3 referred to rotator interval hydro-dilatation.

### Intervention

All patients had diagnostic ultrasonography of the shoulder done by a trained and expert rheumatologist before intervention. Ultrasonography was done for the shoulder by using general electric logic 5 device and a linear high-frequency probe (7.5–12 MHz).

**Fig. 2:**
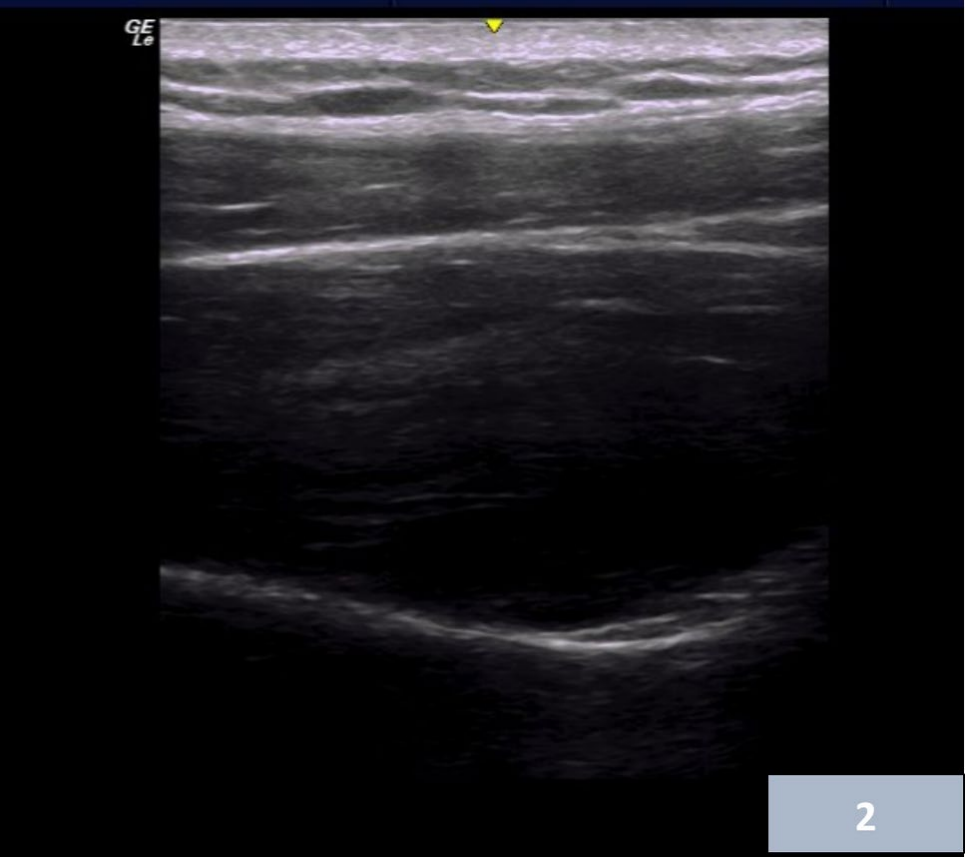
Suprascapular notch and suprascapular ligament superiorly

The first group received an ultrasonography-guided suprascapular nerve block. With the patient in a sitting position, the ultrasound lying anterior to the patient, and the examiner behind the patient, the probe is placed just superior and parallel to the spine of the scapula. Moving the probe slightly laterally, the scapular notch region is recognized, with the suprascapular artery and nerve, along with the transverse scapular ligament. The skin is sterilized with antiseptic, and a 20 gauge spinal needle is inserted in plane with ultra-sonographic guidance, with a direction of slightly from medial to lateral, towards the suprascapular notch. After the needle tip was identified at the suprascapular notch on the sonogram, aspiration was done first to avoid intra-arterial injection. After the nerve was identified, 10 mL of 0.5% lidocaine was injected. A successful intervention is confirmed by the elevation of the transverse scapular ligament (Fig. 2).

**Fig. 3:**
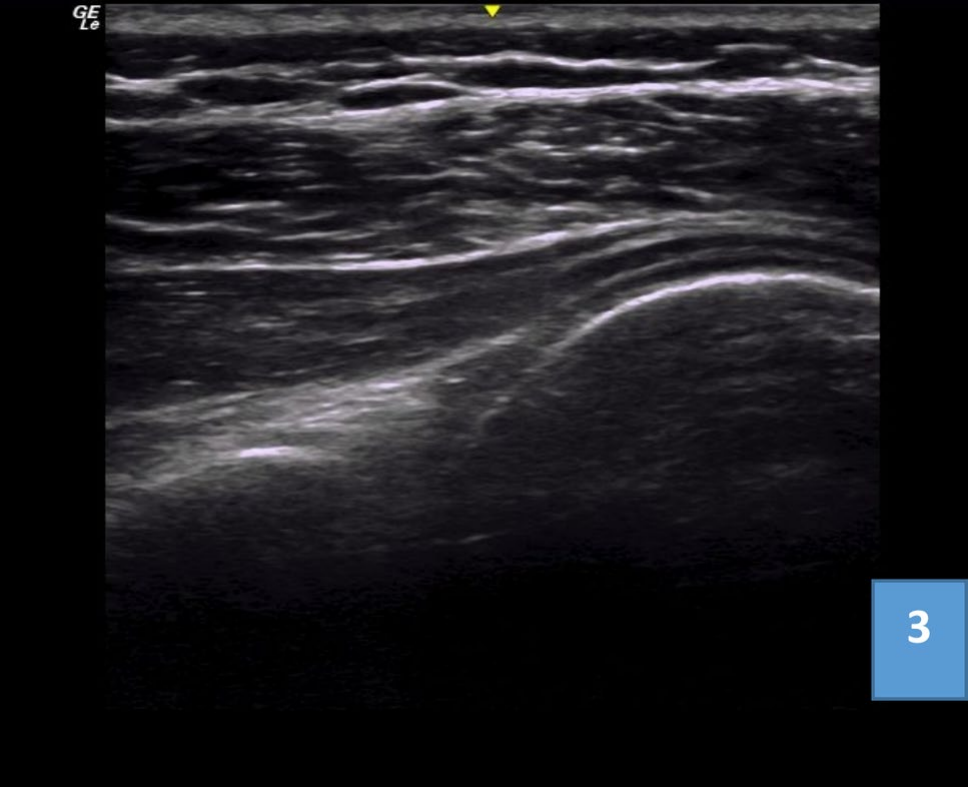
Posterior ultra-sonographic scan of shoulder showing posterior portion of head of humerus, infraspinatus muscle, and posterior joint recess

The second group received intra-articular hydro-dilatation. With the patient in a side-lying position with the shoulder to be injected on top and the ipsilateral arm is resting on a pillow, and the probe placed parallel to the longitudinal axis of the infraspinatus muscle. This position views the posterior portion of the head of the humerus, the posterior glenoid fossa, and the glenoid labrum. After sterilizing the skin, a 20 gauge spinal needle is inserted in plane with ultra-sonographic guidance from medial to lateral targeting the intraarticular space between the humeral head and the glenoid labrum; once the needle reaches the target, first, lidocaine (10 mL 1%) is injected in the glen humeral joint followed by 20 mL of sterile water, slowly to allow acceptance of fluid into the capsule. A successful injection is confirmed by recognizing joint capsule distention and separation (Fig. 3).

**Fig. 4:**
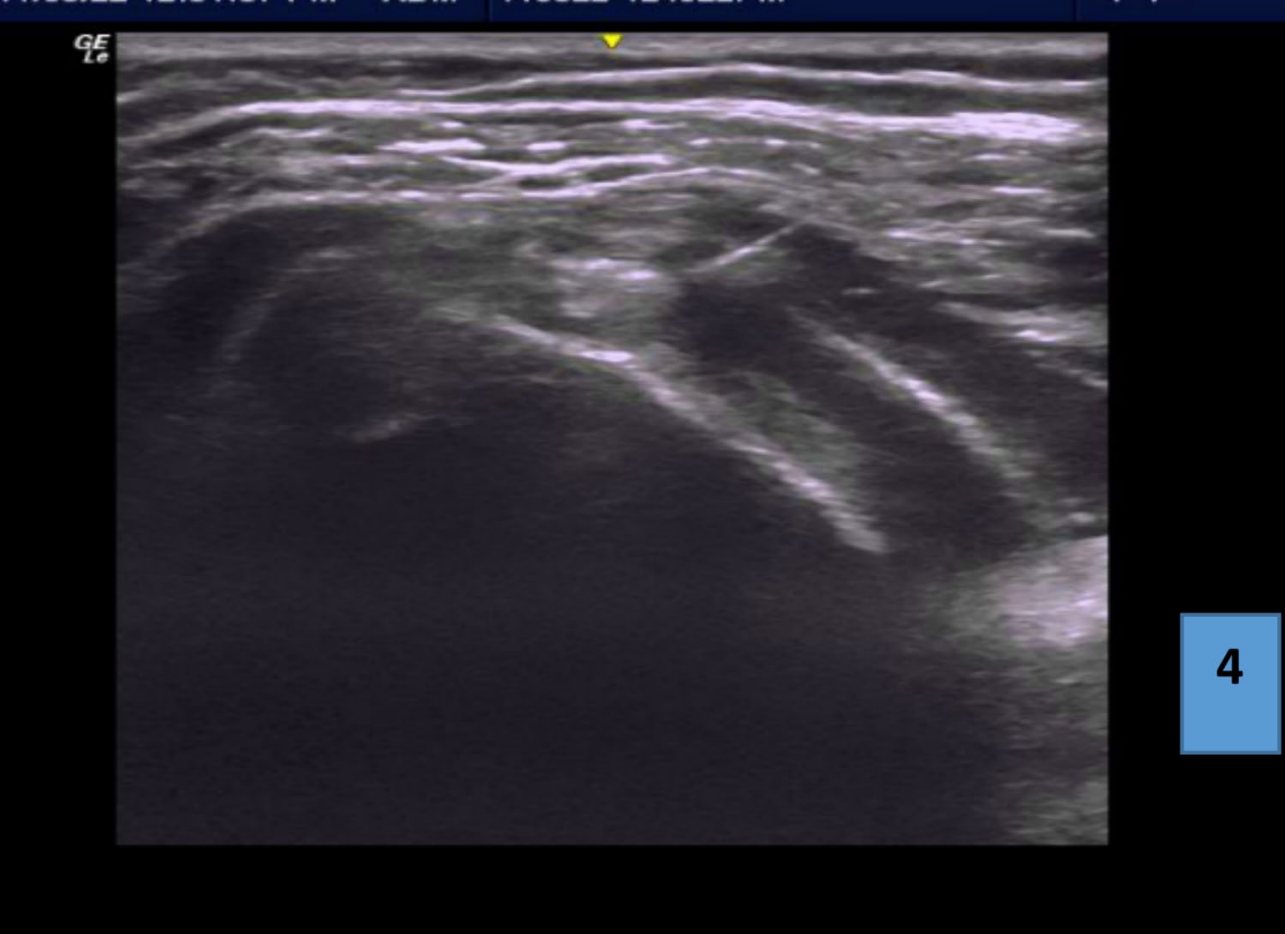
The rotator interval with the biceps tendon in the middle surrounded by the coracohumeral ligament superiorly, subscapularis, and supraspinatus on both sides and showing needle insertion

The third group received shoulder interval hydro-dilatation. With the patient in a sitting position, the affected shoulder is closer to the examiner, and the ipsilateral arm is rotated behind the back. The probe is placed on the anterior shoulder lateral to the coracoid process; this position will show a longitudinal axis view of shoulder interval with the biceps tendon in the middle surrounded by coraco-humeral ligament, and subscapularis and supraspinatus on both sides. After sterilization of the skin, a 20-gauge, 1.75-inch needle is inserted in an in-plane approach from the lateral end of the probe. The needle is inserted deep into the coracohumeral ligament. Once the needle reaches the target, first, lidocaine (10 mL 1%) is injected in the shoulder interval followed by 20 mL of sterile water slowly. A successful injection is confirmed by visualizing peritendinous distention and separation (Fig. 4)**.**

### Outcome measures

Clinical and functional assessment of patients was done at baseline, right after intervention, and 3 months after intervention, Since adhesive capsulitis is a painful condition characterized by restriction of movement, we believed that visual analogue scale (VAS) and range of motion would be essential to assess clinical progression besides being easy and rapid tests that can be applied at multiple follow-ups.

So, our primary outcomes included:

Visual analogue scale (VAS): VAS was used to measure pain severity on a scale from 0 to 100. VAS can be presented in several ways; the simplest VAS is a straight horizontal line of fixed length, usually 100 mm. The ends are defined as the extreme limits of pain orientated from the left (worst) to the right (best) [14].

Range of motion: The range of motion of the shoulder joint as regard: flexion, abduction, and internal and external rotation were measured by the investigator by using a hand-held goniometer [15].

Shoulder pain and disability index (SPADI): It is a questionnaire that measures both pain and disability of the shoulder. It consists of 13 items: a 5-item subscale assessing pain and an 8-item subscale assessing disability. Each item is presented on a scale of 0 to 10, and the patient is asked to circle the number that best describes his pain and disability. Each subscale is summed into a score out of 100. A mean is taken of the two subscales, and a total score out of 100 is given; a higher score indicates greater impairment or disability [16].

In their study to choose the best outcome measures for shoulder pathology, SPADI was recommended by Padua, R., et al., as an assessment tool for shoulder pathological conditions with pain and stiffness [17].

Secondary outcomes: During the study, we recorded three patients with bilateral adhesive capsulitis who showed bilateral improvement with a unilateral suprascapular nerve block.

### Complication

We reported two cases who suffered from vasovagal attacks; both patients had suprascapular nerve block.

**Statistical analysis**: The findings were tabulated, and the results were statistically analyzed using Statistical Package for Social Sciences (SPSS). Quantitative data were presented by suitable parameters such as mean, median, and standard deviation. Comparisons between groups of data were performed using the ANOVA test for continuous variables and the chi-square test for categorical data. The *p*-value was considered significant if less than 0.05.

### Ethical statement

This work was done in accordance with the declaration of Helsinki and after receiving ethical approval from the research ethics committee at Faculty of Medicine in Sohag University and the approval number was Soh-Med-21–05-04.

Informed consent was obtained from all patients prior to performing the procedure.

## Results

The study included 50 patients; four of them had bilateral shoulder injections. We had 20 suprascapular nerve block, 19 intraarticular hydro-dilatation, and 15 rotator interval hydro-dilatation. The mean age of the study in the three groups was (45.40 years vs. 52.05 years vs. 45.73 years) with no significant difference between the three groups (*p* = 0.109). Female participants in the three groups were (70% vs. 78.9% vs. 66.7%; *p* = 0.702) with no significant difference between them. Mean disease duration was 32.25 months in the first group, 33 months in the second group, and 29.47 in the third, with no significant difference between groups (*p* = 0.959). Diabetic patients constituted 20% of the first group, 15.8% of the second group, and 13.3% of the third group; there was no significant difference between the three groups (*p* = 0.86).

Mean duration of the maneuver (the time consumed since needle insertion until needle withdrawal) was 57 s in the first group, 02 m:07 s in the second group, and 03 m:03 s in the third group showing highly significant difference between three groups (*p*-value < 0.0001). Revealing that rotator interval hydro-dilatation was the most time-consuming, while suprascapular nerve block was the least.

Comparison between three groups as regard pain during the maneuver on VAS scale showed highly significant difference between them, the mean VAS score in the first group was 33.16, in the second group was 44, and in the third group was 60 (*p*-value < 0.0001) showing that rotator interval was the most painful procedure, while, again, suprascapular nerve block was the least painful.

A baseline comparison between the three groups revealed no significant difference between them whether SPADI score (*p* = 0.199), range of motion (*p* = 0.2 for flexion, 0.236 for abduction, 0.118 for internal rotation, and 0.842 for external rotation), or pain on VAS score (*p* = 0.326). The first follow-up comparison also showed no significant difference between the three groups for SPADI (*p* = 0.688), for range of motion (*p* = 0.900 for flexion, 0.789 for abduction, 0.104 for internal rotation, 0.356 for external rotation), and for pain on VAS score (*p* = 0.682) The second follow-up comparison also showed no significant difference: for SPADI (*p* = 0.547), for range of motion (*p* = 0.362 for flexion, 0.412 for abduction, 0.740 for internal rotation and 0.872 for external rotation), and for VAS score (*p* = 0.865).

The outcomes of the suprascapular nerve block group are presented in Table 1 showing highly significant difference at 0 vs. 1 comparison (baseline vs. first follow-up), and at 0 vs. 2 comparison (baseline vs. second follow-up) in most parameters and a nonsignificant difference at 0 vs. 2 comparison of internal rotation range of motion.

**Table 1:** Outcomes of the suprascapular nerve block group

	Baseline (0)	First follow-up (1)	Second follow-up (2)	*p* value 0 vs. 1	*p* value 0 vs. 2
Pain at worst	8.30 ± 2.70	4.55 ± 2.46	4.15 ± 2.87	< 0.001 (HS)	< 0.001 (HS)
Pain when lying on affected side	7.95 ± 2.63	4.25 ± 2.67	4.15 ± 3.18	< 0.001 (HS)	< 0.001 (HS)
Pain when reaching for something high	7.60 ± 3.52	3.90 ± 2.73	3.85 ± 3.12	< 0.001 (HS)	< 0.001 (HS)
Pain on touching back of neck	7.10 ± 3.42	3.50 ± 3.30	3.85 ± 3.53	< 0.001 (HS)	< 0.001 (HS)
Pain on pushing	7.55 ± 2.82	4.85 ± 3.48	4.50 ± 3.61	0.001 (S)	< 0.001 (HS)
Disability on washing hair	6.85 ± 3.33	4.10 ± 3.52	3.85 ± 3.88	< 0.001 (HS)	< 0.001 (HS)
Disability on washing back	9.15 ± 1.39	6.15 ± 3.60	6.05 ± 3.89	< 0.001 (HS)	< 0.001 (HS)
Difficulty of putting on a shirt	6.80 ± 3.07	4.60 ± 3.52	3.60 ± 3.68	0.001 (S)	0.001 (S)
Difficulty of buttoning a shirt	3.75 ± 3.55	2.25 ± 3.21	1.85 ± 2.98	0.007 (S)	0.003 (S)
Difficulty of putting on pants	4.70 ± 3.10	2.35 ± 2.82	1.85 ± 2.70	< 0.001 (HS)	< 0.001 (HS)
Placing object on high shelf	8.25 ± 2.90	4.25 ± 3.51	3.50 ± 3.07	< 0.001 (HS)	< 0.001 (HS)
Carrying heavy object 1 pounds	8.50 ± 1.70	5.30 ± 3.16	5.55 ± 3.22	< 0.001 (HS)	< 0.001 (HS)
Remove something of back pocket	7.25 ± 2.81	3.90 ± 3.31	4.50 ± 3.72	< 0.001 (HS)	0.001 (S)
ROM flexion	112.37 ± 46.97	150.50 ± 37.76	140.50 ± 41.10	< 0.001 (HS)	0.002 (S)
ROM abduction	116.25 ± 46.02	148.75 ± 36.05	137.50 ± 42.16	< 0.001 (HS)	0.004 (S)
ROM internal rotation	58.50 ± 19.81	67.50 ± 12.93	62.50 ± 17.73	0.020 (S)	0.330 (NS)
ROM external rotation	35.25 ± 27.31	54.50 ± 30.86	52.25 ± 32.30	< 0.001 (HS)	0.001 (S)
VAS (on 100 score)	89.5 ± 18.49	48.00 ± 27.45	41.50 ± 28.70	< 0.001 (HS)	< 0.001 (HS)

The outcomes of supra scapular nerve block group showed highly significant difference at 0 vs. 1comparison (baseline vs. first follow-up) and at 0 vs. 2 comparison (baseline vs. second follow-up) in most parameters. There was a non-significant difference at 0 vs. 2 comparison of internal rotation range of motion (58.50 ± 19.81 vs. 62.50 ± 17.73; *p* = 0.330)

*HS* highly significant, *S* significant, *NS* non-significant, *ROM* range of motion

The comparison of outcomes of the intraarticular hydro-dilatation is presented in Table 2 showing highly significant difference at 0 vs. 1 comparison and at 0 vs. 2 comparison in most parameters. A nonsignificant difference was found at 0 vs. 1 comparison of internal rotation**.**

**Table 2:** Outcome of the intra-articular hydro-dilatation group

	Baseline (0)	First follow-up (1)	Second follow-up (2)	*p* value 0 vs. 1	*p* value 0 vs. 2
Pain at worst	8.47 ± 2.53	3.84 ± 3.40	4.05 ± 3.88	< 0.001 (HS)	< 0.001 (HS)
Pain when lying on affected side	8.26 ± 2.68	4.00 ± 3.67	4.79 ± 4.29	< 0.001 (HS)	< 0.001 (HS)
Pain when reaching for something high	8.32 ± 2.79	2.89 ± 3.46	3.58 ± 3.55	< 0.001 (HS)	< 0.001 (HS)
Pain on touching back of neck	7.95 ± 2.55	3.26 ± 3.51	3.37 ± 3.79	< 0.001 (HS)	< 0.001 (HS)
Pain on pushing	7.79 ± 2.80	4.37 ± 3.98	3.58 ± 3.75	< 0.001 (HS)	< 0.001 (HS)
Disability on washing hair	7.26 ± 2.86	4.00 ± 3.04	3.16 ± 3.73	< 0.001 (HS)	< 0.001 (HS)
Disability on washing back	8.95 ± 2.55	5.11 ± 3.48	5.63 ± 3.72	< 0.001 (HS)	< 0.001 (HS)
Difficulty of putting on a shirt	7.58 ± 2.52	3.84 ± 3.01	4.00 ± 3.95	< 0.001 (HS)	< 0.001 (HS)
Difficulty of buttoning a shirt	4.68 ± 3.11	1.74 ± 2.58	2.11 ± 3.46	< 0.001 (HS)	0.003 (S)
Difficulty of putting on pants	5.16 ± 3.22	2.21 ± 2.57	1.63 ± 2.83	< 0.001 (HS)	< 0.001 (HS)
Placing object on high shelf	8.32 ± 2.58	3.84 ± 3.76	3.79 ± 3.84	< 0.001 (HS)	< 0.001 (HS)
Carrying heavy object 1 pounds	9.00 ± 1.63	4.42 ± 3.64	5.16 ± 3.47	< 0.001 (HS)	< 0.001 (HS)
Remove something of back pocket	7.47 ± 2.70	4.47 ± 3.10	4.47 ± 3.41	0.001 (S)	< 0.001 (HS)
ROM flexion	94.21 ± 32.54	154.21 ± 30.43	142.63 ± 40.53	< 0.001 (HS)	< 0.001 (HS)
ROM abduction	97.89 ± 30.84	147.37 ± 29.03	142.11 ± 41.58	< 0.001 (HS)	< 0.001 (HS)
ROM internal rotation	44.74 ± 19.33	56.58 ± 19.65	66.32 ± 17.71	0.068 (NS)	0.012 (S)
ROM external rotation	39.47 ± 23.39	66.32 ± 22.41	57.37 ± 29.03	< 0.001 (HS)	0.020 (S)
VAS (on 100 score)	84.21 ± 26.73	41.58 ± 33.71	38.95 ± 36.95	< 0.001 (HS)	< 0.001 (HS)

The comparison of outcomes of the intraarticular hydro-dilatation group showed only a non-significant difference at 0 vs. 1 comparison of internal rotation (44.74 ± 19.33 vs. 56.58 ± 19.65; *p* = 0.068)

*HS* highly significant, *S* significant, *NS* nonsignificant, *ROM* range of motion

The comparison of outcomes of the rotator interval hydro-dilatation group is presented in Table 3 showing highly significant difference at 0 vs. 1 comparison and at 0 vs. 2 comparison in most parameters. A nonsignificant difference was found at 0 vs. 2 comparison of internal rotation and external rotation.

**Table 3:** Outcome of the shoulder interval hydro-dilatation group

	Baseline (0)	First follow-up (1)	Second follow-up (2)	*p* value 0 vs. 1	*p* value 0 vs. 2
Pain at worst	9.47 ± 0.92	3.67 ± 2.69	3.53 ± 1.89	< 0.001 (HS)	< 0.001 (HS)
Pain when lying on affected side	9.27 ± 1.10	3.87 ± 3.14	3.47 ± 2.56	< 0.001 (HS)	< 0.001 (HS)
Pain when reaching for something high	8.87 ± 1.77	3.53 ± 3.04	3.40 ± 2.90	< 0.001 (HS)	< 0.001 (HS)
Pain on touching back of neck	8.47 ± 2.00	3.07 ± 2.60	3.60 ± 3.40	< 0.001 (HS)	< 0.001 (HS)
Pain on pushing	8.47 ± 2.92	4.00 ± 2.83	3.53 ± 3.11	< 0.001 (HS)	< 0.001 (HS)
Disability on washing hair	7.93 ± 2.46	2.87 ± 2.56	2.40 ± 2.64	< 0.001 (HS)	< 0.001 (HS)
Disability on washing back	9.53 ± 1.36	6.33 ± 2.99	4.13 ± 2.95	< 0.001 (HS)	< 0.001 (HS)
Difficulty of putting on a shirt	8.07 ± 2.76	3.40 ± 3.09	2.20 ± 2.73	< 0.001 (HS)	< 0.001 (HS)
Difficulty of buttoning a shirt	6.07 ± 3.96	1.87 ± 2.33	1.33 ± 2.41	< 0.001 (HS)	0.001 (S)
Difficulty of putting on pants	6.87 ± 3.02	1.87 ± 2.17	1.00 ± 2.00	< 0.001 (HS)	< 0.001 (HS)
Placing object on high shelf	8.87 ± 1.60	2.67 ± 2.09	2.27 ± 2.44	< 0.001 (HS)	< 0.001 (HS)
Carrying heavy object 1 pounds	9.07 ± 1.49	4.20 ± 2.65	4.13 ± 3.27	< 0.001 (HS)	0.001 (S)
Remove something of back pocket	8.13 ± 2.39	2.67 ± 2.44	3.73 ± 3.65	< 0.001 (HS)	0.001 (S)
ROM flexion	98.67 ± 22.31	155.33 ± 30.21	158.00 ± 28.34	< 0.001 (HS)	< 0.001 (HS)
ROM abduction	100.00 ± 25.36	154.67 ± 29.73	155.33 ± 33.14	< 0.001 (HS)	< 0.001 (HS)
ROM internal rotation	52.33 ± 22.70	64.00 ± 14.04	62.00 ± 19.71	0.007 (S)	0.131 (NS)
ROM external rotation	39.33 ± 23.75	61.00 ± 20.55	54.00 ± 31.13	0.001 (S)	0.052 (NS)
VAS (on 100 score)	94.67 ± 9.16	40.00 ± 25.07	36.00 ± 19.20	< 0.001 (HS)	< 0.001 (HS)

The comparison of outcomes of rotator interval hydro-dilatation group showed a non-significant difference at 0 vs. 2 comparison of internal rotation and external rotation (52.33 ± 22.70 vs. 62.00 ± 19.71; *p* = 0.131 and 39.33 ± 23.75 vs. 54.00 ± 31.13; *p* = 0.052)

*HS* highly significant, *S* significant, *NS* nonsignificant, *ROM* range of motion

And total SPADI score comparison between the three is presented in Table 4 showing no significant difference at baseline, first follow-up, and second follow-up. However, there is a highly significant difference in each group comparison before and after intervention.

**Table 4:** SPADI score in the three groups

		Suprascapular nerve block	Intra-articular hydro-dilatation	Shoulder interval hydro-dilatation	*p* value
Pain score	Baseline (0)	77.00 ± 25.43%	81.16 ± 24.26%	89.07 ± 13.18%	0.290 (NS)
	First visit (1)	39.20 ± 27.81%	36.74 ± 32.89%	36.00 ± 25.63%	0.942 (NS)
	Second visit (2)	41.20 ± 30.53%	39.32 ± 36.05%	34.27 ± 23.19%	0.800 (NS)
*p* values of pain score	0 vs. 1	< 0.001 (HS)	< 0.001 (HS)	< 0.001 (HS)	-
	0 vs. 2	< 0.001 (HS)	< 0.001 (HS)	< 0.001 (HS)	-
	1 vs. 2	0.755 (NS)	0.707 (NS)	0.857 (NS)	-
Disability score	Baseline (0)	68.56 ± 20.76%	72.96 ± 18.19%	80.58 ± 18.43%	0.196 (NS)
	First visit (1)	41.71 ± 26.39%	36.43 ± 25.26%	31.50 ± 19.26%	0.468 (NS)
	Second visit (2)	38.70 ± 28.96%	36.99 ± 30.51%	26.55 ± 23.56%	0.416 (NS)
*p* values of disability score	0 vs. 1	< 0.001 (HS)	< 0.001 (HS)	< 0.001 (HS)	-
	0 vs. 2	< 0.001 (HS)	< 0.001 (HS)	< 0.001 (HS)	-
	1 vs. 2	0.528 (NS)	0.894 (NS)	0.485 (NS)	-
Total SPADI score	Baseline (0)	71.90 ± 21.02%	76.12 ± 19.30%	83.83 ± 16.20%	0.199 (NS)
	First visit (1)	40.74 ± 25.78%	36.98 ± 27.68%	33.26 ± 21.13%	0.688 (NS)
	First visit (2)	39.63 ± 28.46%	37.51 ± 31.53%	29.42 ± 21.80%	0.547 (NS)
*p* values of total SPADI score	0 vs. 1	< 0.001 (HS)	< 0.001 (HS)	< 0.001 (HS)	-
	0 vs.. 2	< 0.001 (HS)	< 0.001 (HS)	< 0.001 (HS)	-
	1 vs. 2	0.822 (NS)	0.915 (NS)	0.623 (NS)	-

The comparison of pain score, disability score, and total SPADI score between the three groups showed no significant difference at baseline, first follow-up, and second follow-up. However, there is a highly significant difference in each group comparison before and after intervention

*HS* highly significant, *S* significant, *NS* non-significant, *SPADI* shoulder pain and disability index

## Discussion

There are various treatment options available for adhesive capsulitis, including both non-operative and operative approaches. These modalities are generally considered safe and effective for managing pain in the shoulder joint. However, there is currently no high-level evidence to demonstrate the superiority of one treatment modality over another [18, 19].

In our study, we aimed to compare the outcomes of three different treatment modalities: suprascapular nerve block (SSNB), intraarticular hydro-dilatation, and rotator interval hydro-dilatation. We assessed 50 patients, dividing them into three groups based on the type of intervention they received. The evaluation criteria included pain levels measured by the visual analog scale (VAS), the shoulder pain and disability index (SPADI) score, and passive range of motion.

Our findings revealed that all patients experienced a significant improvement in both pain levels and range of motion. However, there was no significant difference between the three treatment groups. Notably, in the SSNB group, patients demonstrated a rapid and sustained improvement in pain, with a nonsignificant improvement in internal rotation range of motion at 12 weeks. Given that pain plays a crucial role in the pathology of adhesive capsulitis, we hypothesized that by targeting pain through suprascapular nerve block, we could break the cycle of limited range of motion and shoulder joint dysfunction. Our results supported this assumption, as SSNB had a significant impact on both pain reduction and range of motion improvement, with a more pronounced effect on pain.

In the group receiving intraarticular hydro-dilatation, patients experienced delayed improvement in internal rotation, but they demonstrated sustained improvement in pain and range of motion in all directions at 12 weeks. This may be attributed to the fact that hydro-distension not only breaks adhesions but also provides analgesic action through the local anesthetic used.

Patients in the group of rotator interval hydro-dilatation showed rapid improvement in pain and range of motion and non-significant improvement in internal rotation and external rotation at 12 weeks. Revealing that suprascapular nerve block and rotator interval hydro-dilatation may have less sustained effect as regard range of motion.

Interestingly, the study also found that the time consumed in each procedure was shortest with a suprascapular nerve block, while it was longest with rotator interval hydro-dilatation.

Additionally, patients reported that rotator interval hydro-dilatation was the most painful technique. This could explain why the posterior intraarticular group achieved better results compared to the rotator interval group, since the latter procedure was less tolerable by the patients, which occasionally forced the operator to inject less amount of fluid, which may have affected the long-term outcomes.

During the study, we recorded three patients with bilateral adhesive capsulitis who showed bilateral improvement with a unilateral suprascapular nerve block. The three patients had cervical spondylosis. This may need further studies with a bigger number of cases and a control group in the future.

The subgroup analysis of Wu et al.‘s study also compared the effectiveness of different approaches to hydro-dilatation, including the anterior and posterior approaches. Interestingly, no significant difference in effectiveness was observed between these approaches. However, the study suggested using the posterior approach with ultrasound guidance due to its easy visualization of the joint. This aligns with our findings that both intraarticular and rotator interval hydro-dilatation had similar outcomes in terms of pain and range of motion improvement. However, intraarticular hydro-dilatation was found to be easier, less time-consuming, less painful for the patient, and had a more sustained effect [20].

In a randomized controlled trial conducted by Parashar et al., three treatment approaches were compared for frozen shoulder: suprascapular nerve block followed by rehabilitation, intraarticular steroid injection followed by rehabilitation, and rehabilitation alone. The study findings indicate that suprascapular nerve block, when combined with non-invasive rehabilitation, is both safe and effective in improving pain and range of motion in patients with frozen shoulder. These results align with our own findings.

Interestingly, the study clearly demonstrates that suprascapular nerve block has a superior effect in reducing pain and disability compared to non-invasive rehabilitation alone or in combination with shoulder intraarticular steroid injection. This suggests that the nerve block plays a significant role in pain management and functional improvement for patients with frozen shoulder.

Furthermore, the study provides an explanation for the observed improvement in range of motion. It attributes this improvement to the effective relief of pain achieved through the suprascapular nerve block. By addressing the underlying source of pain, the nerve block allows patients to engage in rehabilitation exercises more comfortably, leading to enhanced range of motion.

Overall, these findings highlight the importance and effectiveness of incorporating suprascapular nerve block in combination with non-invasive rehabilitation for frozen shoulder treatment. This approach not only improves pain and range of motion but also offers potential advantages over other treatment options, such as intraarticular steroid injections. Further research and exploration of this treatment approach are warranted to validate its benefits and optimize patient outcomes [21].

In a captivating study conducted by Dahan et al., the effectiveness of suprascapular nerve block was compared with a placebo. The results revealed a highly significant improvement in pain within the treatment group compared to the placebo group after 1 month. This improvement was measured using the MPQ multidimensional pain descriptors score. However, there was no significant difference in shoulder function between the two groups, and no notable improvement in shoulder range of movement was observed. These findings are intriguing and can be compared with our own study, which showed that suprascapular nerve block is more effective in providing sustained pain relief rather than improving function and range of motion. However, our study did find that suprascapular nerve block has some positive effect on range of motion, although it may be less pronounced than the impact on pain relief [22].

In an exciting systematic review conducted by Lädermann et al., various conservative treatment options for adhesive capsulitis were compared. Their analysis revealed that hydro-dilatation with corticosteroids emerged as a superior choice for short-term pain relief and improvement in range of motion compared to physiotherapy and corticosteroid injections (CSI). Additionally, the review found no significant difference between the anterior and posterior approaches in hydro-dilatation, aligning with our own findings.

Our research, however, took a slightly different approach. We explored the use of hydro-dilatation without corticosteroids and discovered that the posterior approach yielded better results compared to the rotator interval approach. This distinction sets our study apart and provides valuable insights into the potential variations within hydro-dilatation techniques. By considering the collective evidence, it becomes evident that hydro-dilatation, with or without corticosteroids, holds promise as an effective conservative treatment for adhesive capsulitis. The procedure offers substantial pain relief and improves range of motion, making it an attractive option for patients seeking non-surgical interventions [23].

Singhania, S., et al., made a RCT to compare the effect of hydro-distension with SSNB in adhesive capsulitis; their results were similar to ours as they found that both methods were significantly effective. However, hydro-distension exhibited greater improvements in the ROM, VAS score, and Quick DASH score from baseline to 12 weeks [24].

In a captivating study conducted by Hollmann et al., a subgroup of five patients diagnosed with adhesive capsulitis and experiencing significant movement restriction was investigated. These patients were recommended for capsulotomy, a surgical procedure. Fascinatingly, the study revealed that under general anesthesia, all patients experienced a remarkable improvement in passive range of motion, particularly in abduction, compared to their range of motion while awake. This finding strongly supports the hypothesis suggesting the existence of a fear-based brain-induced movement limitation. Furthermore, it bolsters our own hypothesis that pain plays a partial role in restricting motion in adhesive capsulitis. This scientific evidence highlights the complex interplay between psychological factors, such as fear, and physical factors, such as pain, in adhesive capsulitis. The study provides valuable insights into the underlying mechanisms contributing to movement limitation and offers a foundation for further exploration and understanding of this condition. By delving deeper into the intricate relationship between pain, fear, and movement restriction, we can refine our treatment approaches and develop targeted interventions to optimize patient outcomes [25].

This can also explain our interesting finding of three patients experiencing bilateral improvement of adhesive capsulitis after unilateral intervention; we hypothesized that improving mobility and reducing restriction in the treated side might decrease the compensatory guarding and muscle spasm in the contralateral shoulder.

Another hypothesis that may explain this bilateral improvement is that unilateral suprascapular nerve block may have influenced central sensitization. Chronic pain can lead to central sensitization, where the brain becomes more responsive to pain signals. Reducing pain on one side might have helped decreasing this increased sensitivity, thus benefiting both sides [26].

We also hypothesized it could be due to systemic absorption of the anesthetic used as Kim, M., et al., highlighted in their interesting study. As In this retrospective study, they administered ultrasonography-guided intra articular corticosteroid injection to the shoulder with more severe pain in 165 patients with bilateral symptomatic primary adhesive capsulitis who complained of pain and passive range of motion limitation in both shoulders. They then investigated the changes in the numeric rating scale (NRS) score and passive range of motion values throughout an average 6.7-week follow-up. After the injection, the NRS scores and all passive range of motion values significantly improved not only in the injected shoulder but also in the non-injected shoulder. They attributed the improvement in the contralateral joint to the systemic action of the corticosteroid injected in one shoulder after absorption into the synovium of the glenohumeral joint, leading to inflammation reduction in the non-injected shoulder [27]. However we believe this is an interesting finding that may need further studies with a bigger number of cases and a control group in the future for better understanding.

To our knowledge, no study compares the effect of suprascapular nerve block, hydro-dilatation through posterior intraarticular approach and through rotator interval.

Although no statistical difference was present, we believe that the difference is clinically meaningful and would help physicians to tailor the treatment according to patients; condition with posterior intraarticular hydro-dilatation is the best for patients where restriction is the most prominent symptom, suprascapular nerve block is better for patients where pain is the most prominent symptom, and rotator interval hydro-dilatation being the least recommended at all.

Imitations of our study included that assessors were not blinded to patients’ allocation.

## Conclusion

All three modalities under this study showed significant improvement as regard pain, function, and range of motion. However, intraarticular hydro-dilatation gave the best results as regard improvement in pain and range of motion as well as the sustainability of its effect. It is recommended for patients with severe restriction. Suprascapular nerve block is better used in patients where pain is the most predominant symptom, and in patients with bilateral adhesive capsulitis, it is the technique that takes the shortest duration and fast onset of action.

While rotator interval hydro-dilatation is an effective procedure, we found it has less sustained effect as regard range of motion. Besides being the most painful technique, the least tolerable by the patient, and the one that took the longest duration, all these factors make it the least recommended procedure for treatment. Overall, this study emphasize the importance of personalized treatment approaches for adhesive capsulitis and provide valuable insights for optimizing patient outcomes.

## Data Availability

The data that support the findings of this study are available from the corresponding author upon reasonable and justified request. Data will be retained and made available for a period of five years following the date of publication.
